# Retrieval practice enhances learning in real primary school settings, whether distributed or not

**DOI:** 10.3389/fpsyg.2025.1632206

**Published:** 2025-08-08

**Authors:** Laura Franzoi, Veronica Cembrani, Claudio Mulatti, Barbara Treccani

**Affiliations:** Department of Psychology and Cognitive Science, University of Trento, Trento, Italy

**Keywords:** learning strategies for primary school, retrieval practice, testing, spacing, text comprehension, evidence-based strategies, learning in ecological contexts

## Abstract

With the aim of bridging the gap between laboratory studies and real-world learning experiences, this research investigated the effects of combining retrieval and distributed practice in primary school settings. Retrieval and distributed practice were implemented through a testing procedure that provided accuracy feedback on students' retrieval attempts until they achieved 100% correct answers, and a spacing procedure that lengthened the interval between the initial reading of the study topic (complex school-like materials, i.e., history texts) and the subsequent study session, respectively. In line with existing literature, our findings support the advantage of the testing procedure over the simple re-reading of the material for enhancing 5th graders' learning. In contrast, the manipulation of the spacing interval did not yield a significant effect on students' performance, suggesting the need to explore other sustainable approaches to implement distributed practice. Our results have important implications for educational practice, emphasizing the need to spread knowledge of evidence-based strategies to foster students' text comprehension and long-term retention.

## Introduction

Over the past decades, extensive research has aimed to identify the most effective learning strategies, consistently pointing in the same direction: active learning strategies, based on the retrieval of information from memory, yield superior results compared to passive strategies such as re-reading (for a review, see Dunlosky et al., 2013). As described by Roediger and Karpicke (2006), retrieval practice is a cognitive process that entails the deliberate effort to recall previously learned information from memory, thus strengthening the associated memory traces. This active engagement with the study material not only reinforces and consolidates information in long-term memory but also deepens the comprehension of the content: as learners repeatedly recall and apply their knowledge, they create robust mental connections, fostering a stronger and more interconnected understanding of the content. This iterative retrieval process facilitates the transfer of knowledge, enabling learners to put what they have learned into practice (Karpicke, 2017).

Retrieval-based learning can be implemented through testing. In traditional educational settings, tests are typically administered at the end of teaching units, to assess students' knowledge and acquired skills. However, they can serve as a learning tool as well, promoting active retrieval of information from memory and thus facilitating long-term retention (Rawson and Dunlosky, 2011; Roediger and Butler, 2011). Different test formats can be used, including cued-recall tasks (e.g., through flashcards), free-recall tasks, fill-in-the-blank tasks, matching tasks, multiple-choice tasks, short-answer tasks, and short essays (cf. Yang et al., 2021).

Re-reading, instead, has only limited benefits for learning: it can improve overall comprehension but lacks the active processes of re-elaboration and retrieval that are crucial for deeper understanding and long-lasting learning (Callender and McDaniel, 2009; Carpenter et al., 2009). Research has shown that reading a text over and over again results in a false sense of familiarity with the material, leading individuals to overestimate their understanding and retention despite the non-significant gains in memory consolidation (Koriat and Bjork, 2005). This “illusion of competence” leads to faster but less accurate re-readings, thus preventing the strengthening of memory traces (Rothkopf, 1968).

Experimental studies aiming to compare different learning strategies often follow a three-step design. Firstly, the study material (i.e., paired items) is presented to the students. Then, the study session starts, and some items are restudied (re-read), while others are retrieved (e.g., through a cue-recall test). Finally, a test is administered to assess participants' performance on all items. Consistently across studies, performance on the final test shows a significant improvement for the items that were retrieved compared to those that were restudied. This phenomenon, known as the “testing effect” or “test-enhanced learning” (Fazio and Marsh, 2019), has been well documented in previous literature (e.g., Einstein et al., 2012) and highlights the superiority of retrieval practice in promoting deep comprehension and long-lasting retention of information.

To further optimize learning outcomes, it is possible to combine the effects of different learning techniques. Several authors demonstrated that distributing study sessions over time (i.e., distributed practice) yields better retention compared to cramming the study into intensive sessions (for a review, see Wiseheart et al., 2019). The “spacing effect” involves two key factors: distributing study across multiple sessions rather than a single extended one (Kornell, 2009) and increasing the time intervals between the study sessions (Bahrick, 1979). The effect of this latter manipulation is also described as “lag effect” (Dunlosky et al., 2013). By spreading the study sessions over time, students enable better comprehension, consolidation, and retrieval of information, thus promoting long-term retention. Moreover, research has shown that combining retrieval practice with distributed practice can further enhance the benefits of both strategies (Dunlosky and O'Brien, 2020). When retrieval trials are repeated in time and the lags between trials increase, indeed, the testing and spacing effects can occur simultaneously. This combined strategy is called “successive relearning”.

Despite the wide evidence supporting the benefits of retrieval and distributed practice, several studies documented that these strategies are far from being regularly implemented (Roediger and Pyc, 2012). On the contrary, re-reading and cramming remain very popular among students (Blasiman et al., 2017; Karpicke et al., 2009) and are often encouraged by the teachers themselves (Morehead et al., 2016). This discrepancy could be attributed, at least in part, to students‘ (and perhaps teachers') erroneous beliefs and lack of metacognitive awareness regarding the effectiveness of learning strategies (Blasiman et al., 2017; Cembrani et al., 2023; Cohen et al., 2013; Rivers et al., 2022). Students may perceive re-reading and massed learning as easy-to-implement methods, providing a feeling of fluency, or ease, and learners mistake this sense as an indication that the materials have been well learned. In contrast, effective learning strategies such as retrieval and distributed practice require time, practice, effort, and support, and do not provide immediate rewards.

One of the most recent reviews on these topics is that of Carpenter et al. (2022). In line with previously mentioned studies, they state that, although research has revealed that spacing and retrieval practice reliably enhance learning, these strategies are underused by students, possibly due to metacognitive factors such as false beliefs about learning, lack of awareness of effective learning strategies, or the counterintuitive nature of these strategies. Indeed, according to the authors, successful learning requires a carefully planned “learning routine” (knowledge of the right strategies at the right times) and a regular use of that routine. However, learners usually believe that strategies involving effort are less effective for learning. Indeed, even after directly experiencing spacing and retrieval in their own learning, they rated these strategies as less effective than massing and re-reading, respectively. This misperception matters because learners' beliefs about the effectiveness of strategies are related to the use of those strategies. False beliefs about learning could originate from learners' intuitions, experiences, and even formal education, and both raising awareness about learning strategies and allowing students to experience their effectiveness are needed to change these deep-seated assumptions.

The last decades have seen an exponential growth in the number of studies demonstrating the superiority of retrieval practice and distributed practice. However, their applications in educational settings have received comparatively limited investigation (for a recent review, see Agarwal et al., 2021). Studies are traditionally conducted in laboratory settings, often using simple study materials and involving undergraduate students. However, there is an increasing awareness of the need to analyze the effectiveness of these strategies in real-world educational contexts, using school-like materials and targeting different age groups.

In line with this need, a few studies conducted in actual classrooms have shown the superiority of retrieval practice in ecological settings as well (McDaniel et al., 2007; Roediger et al., 2011; McDermott et al., 2014). Moreover, while the majority of studies on the testing effect have involved the learning of relatively simple material, such as new vocabulary (e.g., Goossens et al., 2014) and word lists (cf. Carpenter, 2012), Carpenter et al. (2009) and Karpicke and Aue (2015) found benefits of retrieval practice in the retention of complex contents too, suggesting that such benefits are not restricted to basic learning tasks but can successfully apply to more challenging ones. Additionally, studies investigating the retrieval practice benefits across different educational levels have shown promising results. Although most evidence still comes from university students, research by, for example, Goossens et al. (2014), Carpenter et al. (2009), Karpicke et al. (2014, 2016), Dobson and Linderholm (2015), Fazio and Marsh (2019), (Lipowski et al. (2014)), and Moreira et al. (2019) support the effectiveness of retrieval practice across primary and secondary education levels beyond the higher ones.

While these studies provide valuable insights into the potential of retrieval practice in educational settings, further research is needed to explore the full potential of this procedure, including its optimal implementation, its effects on different student populations, and its combination with other effective learning strategies.

Focusing on primary school students is a valuable approach, as acquiring effective learning strategies at that age can have long-lasting benefits throughout their educational journey. However, conducting experiments with children in real educational settings proves challenging due to the need to both choose an appropriate study design and motivate participants to actively engage with the tasks, which is a necessary condition for active learning strategies (Roediger and Pyc, 2012). To address these challenges, it is crucial to carefully choose appropriate tasks for both the study session and the final evaluation. Therefore, we relied on previous literature as well as on the expertise of the teachers, thus ensuring that the tasks were aligned with the students' age and school level.

The existing evidence suggests that retrieval practice is more effective when implemented through tests that require generative responses, such as free recall or short answers, rather than less demanding ones like multiple-choice. Long-term retention, indeed, is enhanced when open-question or fill-in-the-gap tests are used, as the learner's effort to retrieve plays a crucial role (Jaeger et al., 2015). On the contrary, multiple-choice tests primarily assess recognition skills, without an active retrieval and elaboration of the material (Carpenter and DeLosh, 2006; Glover, 1989; Hinze and Wiley, 2011). Tests should therefore be sufficiently demanding, but a balance between difficulty and executability is needed, especially when young students are involved. The benefits of retrieval practice, indeed, rely on the possibility for students to overcome the learning challenge (Bjork and Bjork, 2011). For example, Karpicke et al. (2014) highlighted the need for guided retrieval practice methods for young children, as they tend to struggle with methods, such as unsupported free recall and concept mapping activities, that are known to be effective with university students.

The same applies to distributed practice, as incorporating this strategy into students' study routines can prove extremely challenging, particularly for younger learners (Son and Simon, 2012). Therefore, as suggested by Dunlosky et al. (2013), instead of using multiple identical short study sessions spaced by long intervals and comparing them with a fully massed condition (i.e., no interval between sessions), we decided to manipulate the time gap between two different study sessions: the initial reading of the material (which typically reflects how students first engage with a new content) and a subsequent study session (which would be conducted following different procedures), This manipulation aimed to help students implement distributed practice relatively easily—easily enough that they could apply it autonomously even beyond the context of the present study—thus increasing the practicality of this effective learning approach in real educational contexts. Furthermore, we intended to minimize disruptions to the lesson schedule of the participating classes, thus ensuring the ecological validity of our findings.

The effectiveness of retrieval practice is also influenced by the presence of feedback (Pashler et al., 2005). This strategy, indeed, is more beneficial when students retest themselves until they reach 100% accuracy (Dunlosky and O'Brien, 2020; Lipko-Speed et al., 2014), and feedback is helpful as it prevents error preservation (Kang et al., 2007; Vojdanoska et al., 2010). However, the self-correction process based on feedback may pose significant challenges to young students when open-question tests are provided, potentially reducing their motivation and performance. Fill-in-the-gap tests may therefore represent a more appropriate tool since they lead to an easier self-correction process.

The aims of our study were therefore the following:

- To examine whether the benefits of retrieval and distributed practice can be observed in primary school settings when complex and naturalistic materials (part of the history curriculum) are used, and activities are led by teachers during regular classroom lessons. Although studies involving primary school children, conducted in real-life classroom settings, and using complex, naturalistic materials are becoming more common, research that combines all these characteristics remains relatively rare.
- To evaluate the suitability and effectiveness of fill-in-the-gap questions as a self-testing tool for implementing retrieval practice with primary school students.
- To investigate whether the benefits of distributed practice can be observed in primary school settings by implementing a simple protocol involving an initial reading of the material, followed—after one of two relatively short intervals (reasonably feasible within a primary school context)—by a second study session, thereby providing an ecologically valid approach to applying this strategy.

Existing literature offered preliminary evidence suggesting that these specific implementations of the two selected learning procedures might potentially improve long-term retention of study material. Our aim was to verify this hypothesis and assess the actual impact of the two procedures when combined within the particular real-world primary school educational context under investigation.

## Methods

### Participants

Thirty-three children aged between 10 and 11 took part in the experiment. They all attended the 5th grade of an Italian primary school and had an adequate knowledge of the Italian language. The children belonged to two different classes: class 5A (15 students; 5 girls) and class 5B (18 students; 8 girls). The sample size was determined by the volunteer participation of the teachers and their classes. See the Supplementary material for further details regarding participants' recruitment.

### Design

**Figure 1:**
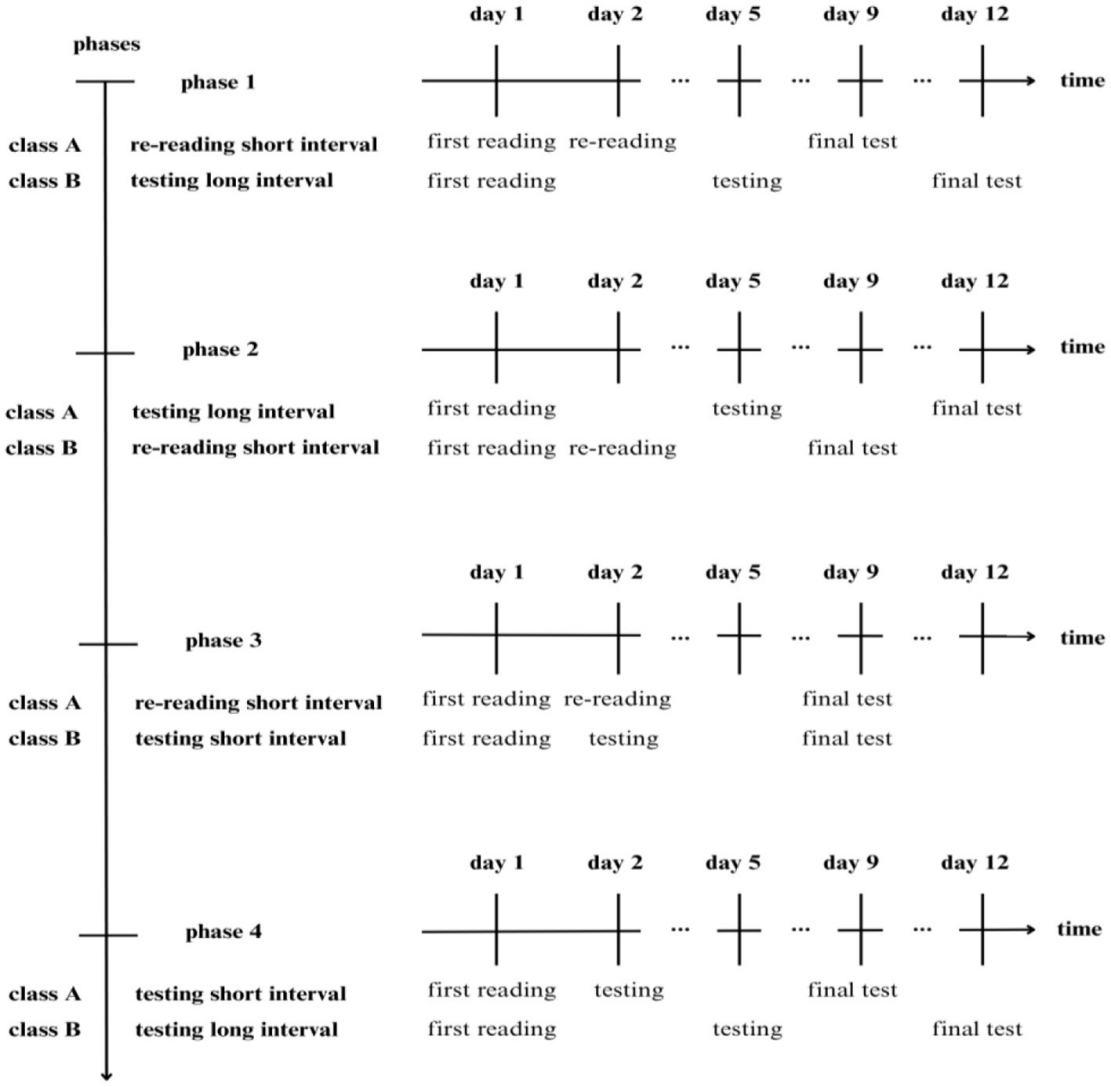
Schematic representation of the experimental procedure and design.

Two variables were manipulated in the study^1^: STRATEGY (Re-reading vs. Testing) and SPACING INTERVAL (Short interval vs. Long interval). STRATEGY was manipulated by instructing participants to use a different learning approach during the study session, whereas SPACING INTERVAL was manipulated by changing the time interval between the initial reading of the material and the subsequent study session, i.e., 1 day (Short interval; i.e., the massed condition) vs. 4 days (Long interval; i.e., the distributed condition). The experiment was organized into 4 phases. In the first two phases, we tested Testing Long interval against Re-reading Short interval. In Phase 1, class 5B was assigned to the Testing Long-interval condition and class 5A to the Re-reading Short-interval condition. In Phase 2, the assignment was reversed: Classes 5B and 5A underwent the Re-reading Short-interval and Testing Long-interval conditions, respectively. In Phase 3, STRATEGY was manipulated (classes 5A and 5B were assigned to the Re-reading and Testing conditions, respectively) while SPACING INTERVAL was held constant (Short interval condition only). In Phase 4, SPACING INTERVAL was manipulated (classes 5B and 5A were assigned to the Long interval and Short interval conditions, respectively) while holding constant STRATEGY (Testing condition only^2^; see Figure 1).

### Materials

In our experiment, we decided to use fill-in-the-gap tests for the study session, being them suitable for both the retrieval and the self-correction processes. For the final evaluation, we opted instead for open-ended questions, as students did not need to self-correct their answers at that stage and as the testing effect persists even when the final test differs from that used during the study session (Kang et al., 2007; Yang et al., 2021). All materials concerned ancient history and were prepared in collaboration with the teachers, following the history program of the classes involved. This alignment with the curriculum and learning goals made the study relevant and valuable within the educational context, thus boosting its ecological validity. The content of the four texts (one for each phase), while different, had been judged as comparable in terms of complexity by the teachers involved and was unfamiliar to the students. The texts ranged in length from 590 to 660 words and covered topics such as “The Ancient Greece” and “The foundation of Rome”. The test for the study session of the Testing conditions consisted of 12 to 14 fill-in-the-gap items, whereas the final evaluation tests (for both Testing and Re-reading conditions) included 12 to 16 open-ended questions. Most of the questions of the four final tests were literal, with only a few inferential ones. An example of a fill-in-the-gap question is “The new king of the Macedon empire was labeled ___ (Magno), meaning ___ (Great)”, whereas that of an open-ended question is “Who was the young descendant of the first Macedon king? (Alessandro Magno)”.

See the Supplementary material for the original versions of the materials (i.e., the texts, the fill-in-the-gap tests used during the study sessions, and the open-ended questions used in the final tests), as well as more details on how they were created.

### Procedure

The experiment took place at school during regular history lessons led by the usual teachers of the students. The study's activities were integrated into the regular classroom routine. The experiment spanned approximately seven weeks, during which each class participated in 12 activity days (three per phase), as shown in Figure 1. Each phase lasted 12 days and began immediately after the previous one, although the intervals between phases may have varied slightly due to the school calendar and holidays. The day of the final test of each phase was not communicated to the students in advance, so that they couldn't prepare for it at home. The experiment had no impact on the final history grade, and students were not evaluated by the teachers for their performance on the final tests.

Each of the four phases of the experiment was structured as follows (see Figure 1). In Step 1, children received the text and were instructed to read it carefully twice. This ‘first reading' step represented the initial presentation of the study material. After 1 day (short interval; i.e., our “massed” condition) or 4 days (long interval; i.e., our “distributed” condition), participants completed Step 2: They re-read the text as many times as they wanted if assigned to the Re-reading condition or completed a fill-in-the-gap test if assigned to the Testing Condition. In the latter case, participants were instructed to complete as many gaps as they could and were then provided with correct answers for self-evaluation. If they left at least one space empty or filled it with the wrong answer, they were required to return the correction sheet to the teachers and attempt to rectify their errors on their own. Subsequently, they could review their answers once more, with only one final opportunity to fill all the gaps correctly. This procedure allowed every student to reach complete accuracy by the end of the session. The entire procedure lasted a maximum of 30 min, which is the same time limit as that of the Re-reading condition. Seven days after Step 2, the final open-question test was administered, and students had 10 min to complete it. See the Supplementary material for a more detailed description of each step of the procedure.

## Results

### Scoring

Data from students who were absent from school at any step of the procedure of a given phase were excluded from the analysis. Therefore, the final sample sizes were 27, 26, and 25 participants (out of the original 33) for the analyses of Phases 1 and 2, Phase 3, and Phase 4, respectively. Final scores were converted to percentage values. See the Supplementary material for further details on the scoring process. Table 1 shows the mean accuracy percentage scores obtained by participants following the different learning procedures tested across the four experimental phases.

**Table 1:** Accuracy percentage scores in the four experimental phases as a function of the learning procedure.

**Experimental phase**	**Learning procedure**	**Mean score(standard deviation)**
Phases 1 and 2	*Testing long* interval	67.0 (21.7) %
	*Re-reading short* interval	48.6 (21.6) %
Phase 3	*Testing* short interval	67.7 (21.4) %
	*Re-reading* short interval	41.3 (25.3) %
Phase 4	Testing *Long* interval	45.9 (22.1) %
	Testing *Short* interval	44.6 (22.8) %

### Phases 1 and 2

**Figure 2:**
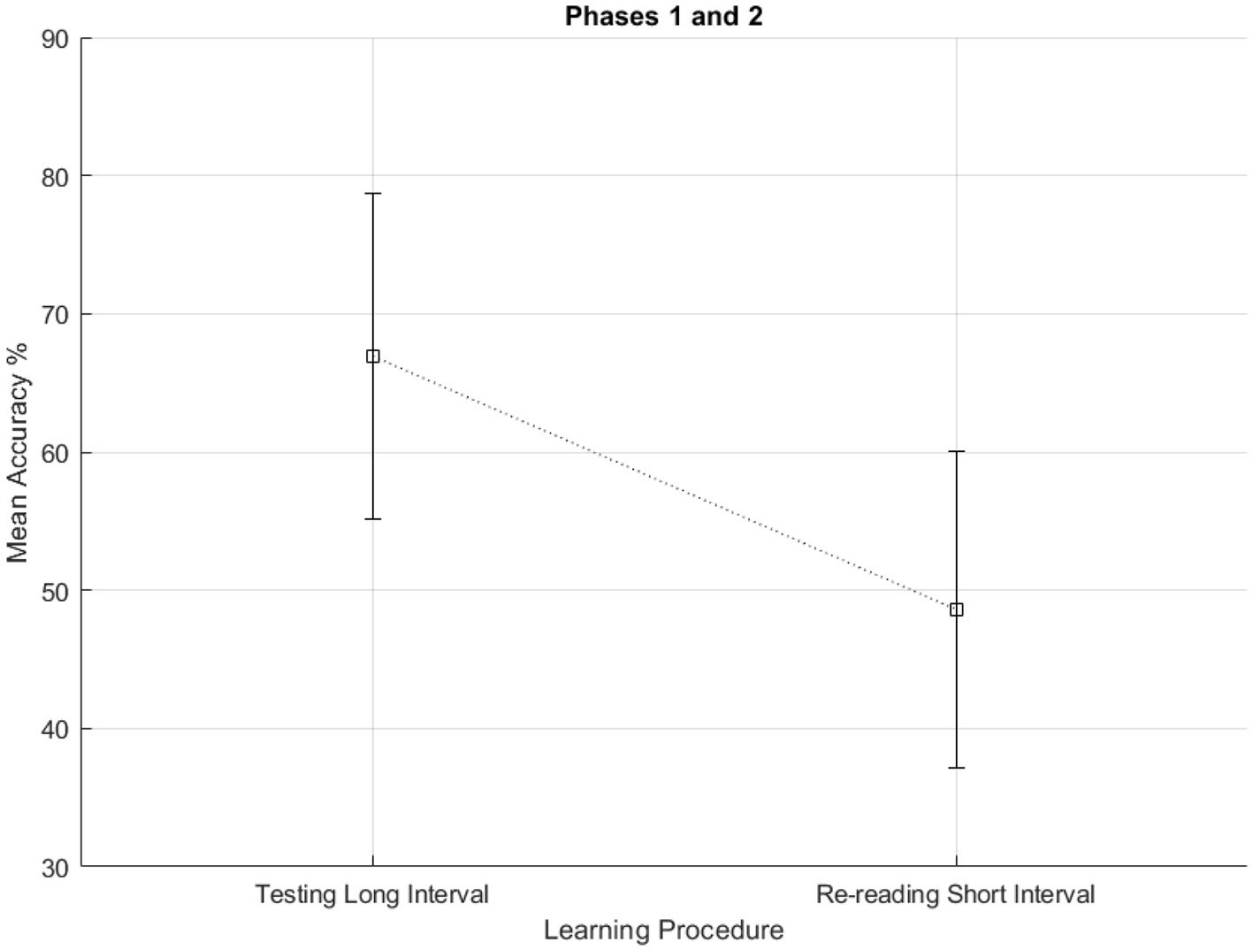
Mean accuracy percentage scores obtained by participants in the two Learning Procedure conditions of Phases 1 and 2. Vertical bars represent 95% confidence intervals.

Results of Phases 1 and 2 were analyzed using a mixed ANOVA, with Learning Procedure (Re-reading Short Interval vs. Testing Long Interval) as a within-subject factor, and Order of Conditions (i.e., which learning procedure was presented first, in Phase 1, and which as second, in Phase 2: Re-reading Short Interval first vs. Testing Long Interval first) as a between-subject factor. The main effect of Learning Procedure proved significant, F_(1, 25)_ = 41.42, *p* < 0.001, η2 = 0.154, indicating that participants' mean accuracy percentage scores in the Testing Long-interval condition were higher than in the Re-reading Short-interval condition (67.0% vs. 48.6%, respectively), as shown in Figure 2. In contrast, neither the main effect of Order nor the interaction of the two factors was significant, both Fs <1. Since the order in which the two learning procedures were assigned was confounded with the class (5A vs. 5B) to which participants belonged, the lack of a significant effect of Order also indicates no significant differences between the two classes. This suggests that the observed effect of the Learning Procedure was genuine and not to be attributed to the specific class of the students. The absence of a significant interaction also indicates that the advantage of the Testing Long-interval condition over the Re-reading Short-interval condition was the same regardless of the order of these conditions, that is, of whether they were assigned to participants in Phase 1 or 2. This also suggests that the specific materials (texts, fill-in-the-gap tests, and final tests) used in the two phases were equivalent in terms of complexity, although the content was different: A difference in material difficulty between the two phases would have increased the difference between Testing Long Interval and Re-reading Short Interval in one order condition and reduced it in the other, resulting in a significant interaction between Order and Learning procedure.

### Phase 3

We performed an ANOVA with STRATEGY (Re-reading vs. Testing) as a between-participants factor. The analysis proved significant, F_(1, 24)_ = 8.2, *p* < 0.01, η2 = 0.254, with participants in the Testing condition outperforming participants in the Re-reading condition (mean accuracy percentage scores: 67.7% and 41.3%, respectively).

### Phase 4

An ANOVA with SPACING INTERVAL (Massed vs. Distributed) as a between-participant factor was performed. The result was not significant, F <1: Participants in the Short-interval condition performed similarly to participants in the Long-interval condition (mean accuracy percentage scores: 44.6% and 45.9%, respectively).

## Discussion

Several studies have demonstrated that retrieval-based learning strategies outperform passive ones (Dunlosky et al., 2013) and that distributing study over time is more successful than cramming (Kornell, 2009). However, most students still tend to use passive techniques (Karpicke et al., 2009) and to mass their study (Son and Simon, 2012). These ineffective behaviors can be attributed to a lack of metacognitive awareness (i.e., students' understanding and monitoring of their learning processes), which leads students to underestimate the advantages of effective learning strategies while overestimating the benefits of less effective ones (Blasiman et al., 2017). This set of evidence leads to important implications in the educational field, emphasizing the need to teach and implement effective learning strategies in real school contexts too (Agarwal et al., 2021).

Our experiment aimed to further analyze two learning strategies that have been shown to be effective, retrieval and distributed practice, by evaluating their effects on primary school students' retention of authentic school material in a real classroom setting. We believe that the primary school years represent a critical period in a student's educational journey. During this phase, children acquire foundational knowledge and develop essential learning habits that can significantly impact their future academic performance. By identifying and implementing effective learning strategies at this stage, students can acquire skills and techniques that will have long-lasting benefits throughout their educational journey and beyond.

Results of the first two phases of our study demonstrated that the Testing Long-interval Procedure leads to better results compared to the Re-Reading Short-interval one. However, as STRATEGY and SPACING INTERVAL were co-manipulated in these phases, we could only evaluate their combined effect.

In Phases 3 and 4, we manipulated the variable STRATEGY, while holding constant SPACING INTERVAL, and the variable SPACING INTERVAL, while holding constant STRATEGY, respectively. We found that testing promotes better learning than re-reading, in line with previous literature. However, in contrast to findings from prior studies, the spacing interval alone (i.e., in Phase 4) did not affect students' performance in our study.

The lack of observed spacing effects in Phase 4 may stem from the specific way in which spacing was manipulated in our experiment. Although the design of Phase 4 mirrors that of a typical study on the spacing effect (cf., Carpenter, 2012), in many previous studies, distributed practice involved studying the same material over multiple short sessions spread out over time, whereas massed practice typically consisted of presenting and re-studying the material on the same day—that is, with a zero time gap between sessions, essentially forming a single extended study session. In our experiment, by contrast, both the massed and distributed conditions comprised two spaced study sessions—the initial reading of the study material and a subsequent study session —with the only difference being the length of the interval between these sessions (i.e., short vs. long: 1 vs. 4 days). This kind of manipulation aimed to minimally affect the regular lesson schedule of the two classes participating in the experiment, thus ensuring the ecological validity of the study, which was one of our main goals. Moreover, if successful, it could have provided a convenient and straightforward method for implementing this effective learning approach in primary school classrooms: two sessions, separated by a reasonable amount of time—only a few days—seem to be a feasible and acceptable way of applying distributed practice. As research indicates, indeed, distributed practice involving multiple short study sessions— particularly when spaced by relatively long intervals — is typically challenging for students to incorporate into their study habits, even when they are aware of its benefits (Blasiman et al., 2017).

It is worth noting that, although most studies have demonstrated the spacing effect as a disadvantage for study sessions with no interval between them (i.e., the proper “massed” condition; Carpenter, 2012) compared to sessions separated by a nonzero delay, previous research had already shown that increasing the spacing between learning sessions enhances long-term retention (e.g., Bahrick, 1979; Dunlosky et al., 2013). For example, Carpenter et al. (2009) found differences in learning outcomes when comparing a spacing interval of one week to a spacing interval of 16 weeks. Our results, however, suggest that the benefits of lengthening the delay between study sessions may be more limited than previously thought and/or dependent on specific conditions. For example, in our case, participants in both SPACING INTERVAL conditions studied the material using the testing strategy, which has been proven to be the most effective one, and this may have masked any potential (additional) benefits of the SPACING INTERVAL. Secondly, it is possible that the interval between the initial presentation of the material and the subsequent study session in the Short-interval condition (i.e., 1 day, our massed condition) was already sufficient to support effective learning. Conversely, the interval in the Long-interval condition (4 days) may not have been sufficiently different from the short one to produce a noticeable difference in performance, or, alternatively, it might have been too long to fully realize its potential effectiveness, as some students may have forgotten part of the content presented in the initial reading session by the time the subsequent study session occurred. Indeed, there is evidence supporting this possibility. For example, Cepeda et al. (2008) found that as the gap between study sessions increases, performance first improves and then gradually declines, suggesting that overly long intervals can be detrimental, probably because they increase the likelihood that students forget critical information from one session to the following one. For a study session to be effective, students must retain a certain amount of information from the previous session. Further studies are needed to uncover optimal strategies for implementing distributed practice in educational contexts and to determine its impact on students' learning outcomes.

Finally, it is also worth mentioning that the mean percentage scores in Phase 4 were lower than those observed in Phases 1, 2, and 3 (see Table 1), which were quite similar to each other. This might suggest that Text 4 was more difficult than the others. Clearly, our aim was to create four texts comparable in complexity. However, in line with our goal to evaluate different learning procedures in a primary school setting through a naturalistic and ecological approach, we relied on teachers' feedback to develop the materials according to the history curriculum of the classes involved. Consequently, each text covered a different topic, and it is possible that one of the texts ended up being more difficult than the others. Upon revisiting Text 4, we observed that, unlike the other texts, it was more expository than narrative in nature (i.e., it contained factual and conceptual information about Roman society, such as the roles and identities of patricians and plebeians), which may have increased its cognitive demands and contributed to the lower scores. Regardless of the reasons that made Text 4 more difficult to process or recall for the students, the generally poorer performance on this text may have obscured potential spacing effects.

Our findings, combined with existing literature, have significant implications for the educational context. One of our primary goals was to provide further evidence of the benefits of retrieval practice in a primary school setting, with the aim of encouraging teachers to adopt this strategy with their students. In fact, testing is rarely used as a technique to enhance classroom learning (Roediger and Pyc, 2012) and it is primarily viewed as a tool to assess students' performance. However, research has demonstrated that it can serve as a powerful learning tool as well (Dunlosky et al., 2013; Karpicke, 2017). Therefore, it is important to start incorporating effective learning strategies into instructional materials and lesson plans. By designing curricula that integrate retrieval practice and other effective strategies, teachers could create a more engaging and productive learning environment for their students, thus enhancing their comprehension and retention of material. Moreover, by introducing effective learning strategies in the classroom, we believe that students will also be more likely to apply them when studying alone.

Moreover, our study highlights the appropriateness of fill-in-the-gap tests as an effective testing technique in a primary school context. Fill-in-the-gap tests have been well described as suitable and successful in evaluating the knowledge and understanding of young learners (Jaeger et al., 2015; Moreira et al., 2019); however, our findings suggest that they also constitute an appropriate format to implement retrieval practice. The use of fill-in-the-gap tests provides several advantages in the educational context. Firstly, these tests offer a more interactive and engaging way for students to demonstrate their comprehension of the topic. By allowing students to recall and apply their knowledge to complete the missing parts of a sentence or passage, fill-in-the-gap tests promote active engagement and deeper processing of the material. Moreover, they provide a targeted assessment of specific concepts. Educators could therefore design these tests to focus on key learning objectives, providing valuable insights into students' grasp of essential content. This may also help them to identify the areas where students struggle most and tailor their instruction accordingly. Furthermore, as they do not require extensive time for completion, fill-in-the-gap tests are suitable for both regular assessments and homework assignments. The simplicity of the format also enables students to use corrective feedback to self-assess their performance and independently identify areas to improve. Therefore, we suggest considering the incorporation of fill-in-the-gap tests into educational practice.

One crucial step towards implementing effective learning strategies is to provide teachers with training and resources to effectively guide their students through the learning process and create a supportive learning environment, thus enhancing learning outcomes. Carpenter and Agarwal (2019) took a step forward in this direction. Moreover, educators could use their knowledge to promote metacognitive awareness among students, helping them develop a deeper understanding of effective learning techniques.

While our study yielded interesting findings, it is important to acknowledge its limitations. Firstly, due to convenience factors, the sample size was small. We could not rely on an a priori power analysis to determine the optimal sample size, since—as it often happens in real-world settings—the sample size was dictated by the context in which the study was conducted. We planned to include, and indeed included, all participants whose teachers had agreed to take part in the study. In this type of research, the goal is often to identify effects of a certain magnitude, which are not only statistically significant but also meaningful in practical terms. For such effects, relatively small samples can sometimes be sufficient, as was the case in our study. Nonetheless, it is undeniable that including more students would have increased the statistical power and potentially allowed for the detection of smaller effects—less relevant from a practical standpoint, but possibly meaningful from a theoretical perspective—such as those that did not reach significance in this study.

Secondly, to minimize disruption to the regular lesson schedule, the experimental design did not involve a complete counterbalancing of the experimental conditions. In fact, we implemented counterbalancing in Phases 1 and 2, but not in Phases 3 and 4. The lack of counterbalancing would have been critical in the absence of equivalence between the two classes involved in the study. We assumed that the two classes were similar since they belonged to the same school, shared the same social context, and had students who were comparable in terms of age and school level. The absence of a significant effect of the between-subjects factor in the analysis of the Phases 1 and 2 data (i.e., the Order of conditions, which also corresponded to the tested class) supports this assumption. However, reversing the assignment of conditions to the two classes in Phases 3 and 4 could have provided more accurate and reliable results.

Finally, it is worth noting that in our study the learning strategies were implemented in the classroom, with students completing the task individually but simultaneously and in the same environment, under the supervision of the teachers. Although this mimics what happens during regular lessons, the results of the study would have had greater generalizability if a condition had also been included in which students applied the strategies separately, each in their own space and without supervision, as they would when studying alone at home. Indeed, there is evidence that performing a task with others—or even simply being in their presence, whether actual or imagined—can influence how the task is carried out (cf., e.g., Sellaro et al., 2020; Zajonc, 1965).

Further research is needed to expand our understanding of retrieval and distributed practice in educational contexts. By exploring how distributed practice could be effectively implemented and integrated into the existing curricula and instructional practices of primary schools, researchers could identify practical suggestions and guidelines for educators. Some recent studies have specifically addressed this aspect. Ortega-Tudela et al. (2021), for example, examined the feasibility and effectiveness of retrieval-based learning in young children when teachers themselves design and implement retrieval activities relating to genuine curriculum content, finding promising results. Additionally, further research could shed light on potential factors that may influence the effectiveness of retrieval and distributed practice, such as instructional approaches, the nature of the study material, and the learning context. Some studies have already been conducted with this aim. For instance, Goossens et al. (2014) investigated the effect of retrieval practice in the context of two different vocabulary learning methods. In their study, primary school students either read and listened to a story in which novel words were embedded and explained by the experimenter, or they read and listened to word pairs, with each novel word presented alongside a familiar synonym. The two methods differed in overall effectiveness (with the word-pair method proving more effective). Retrieval practice was found to be more beneficial than simple re-reading in both cases, even though its benefits in the word-pair condition were somewhat larger than in the story condition.

Another interesting line of research would be to analyze the benefits of retrieval practice compared to those of other learning activities commonly implemented in the classroom. For instance, retrieval practice should be opposed not only to re-reading but also to other effective strategies such as concept mapping (see Moreira et al., 2019). Additionally, it would be interesting to explore the effects of retrieval and distributed practice on less-explored subjects like mathematics, as well as their impact on performance when different text comprehension measures are used (e.g., inference-based questions) and the transfer of the acquired knowledge to other domains (Carpenter, 2012).

Further studies could also examine how different learning strategies affect students with specific learning disabilities or varying levels of achievement. Surprisingly, very few studies have addressed this issue. For example, Karpicke et al. (2016) investigated whether the benefits of retrieval practice were influenced by individual differences in reading comprehension and processing speed in primary school children, but found that its benefits were largely independent of these factors. However, theirs is one of the few studies to explore this topic. The surprising lack of research on retrieval and distributed practice targeting individual differences is noteworthy given the significance of such differences in educational settings (see Moreira et al., 2019).

In conclusion, our study highlights the importance of integrating effective learning strategies into educational practice. The findings confirmed the significant benefits of retrieval-based learning for young students, even when implemented in a real classroom environment. By contributing to the existing knowledge on effective learning strategies, we aim to provide valuable guidance for educators and curricula developers. Based on these findings, teachers should be encouraged to adopt these learning strategies in their classrooms, thus promoting students' metacognitive awareness and fostering the development of effective study habits. This would have a positive impact on students‘ educational journey and, consequently, on their future lives, opening a wider range of opportunities for their personal and academic growth.

## Data Availability

The datasets presented in this study can be found in online repositories. The names of the repository/repositories and accession number(s) can be found below: https://osf.io/d7x96/?view_only=b5e20123b0d545de820ec68e6cfcc2b6.

## Footnotes

^1^As often occurs in real-world educational settings, the study followed a quasi-experimental design: we used pre-existing groups corresponding to the two classes that agreed to collaborate with us.

^2^Due to the limited availability of participant groups (restricted to the two classes that consented to take part in the study), it was not possible to fully cross the four conditions. That is, we could not compare the two levels of the STRATEGY variable at both levels of the SPACING interval variable, nor the two levels of the SPACING variable at both levels of the STRATEGY variable.
