# Supplementary material for: Retrieval practice enhances learning in real primary school settings, whether distributed or not
Source: Front Psychol. 2025 Aug 8;16:1632206. doi: 10.3389/fpsyg.2025.1632206 (PMC12372469; doi:10.3389/fpsyg.2025.1632206)
Supplement: Supplementary file 1 [file Supplementary_file_1.pdf]

# Retrieval Practice Enhances Learning in Real Primary School Settings, Whether Distributed or Not.

## 1. APPENDIX

This section contains further details regarding: Participants recruitment, Creation of the materials, Procedure, Scoring, and some Additional analyses we conducted.

**1.1. Participants recruitment.** Classes were recruited on a voluntary basis. To ensure their involvement, we sent an email to the school administrator requesting the cooperation of the school. The decision to participate in the study was left to the individual choice of the teachers. Prior to the participation of the children in our study, we obtained informed consent from their parents or legal guardians. All students of both classes took part in the experiment. The teachers assigned a unique numerical code to each student, which was used on the answer sheets. This system allowed us to ensure that the data could not be directly linked to a specific student, maintaining the confidentiality of their participation.

**1.2. Creation of the materials.** We prepared four texts with a word length ranging from 590 to 660 and a number of paragraphs ranging from 14 to 19, one for each phase of the experiment. The topic of the texts was ancient history, and they were prepared together with the teachers to be aligned with the 5$^{th}$-grade history program. The texts were new to the participants and were not present in their schoolbooks, to avoid uncontrolled exposure to the experimental materials. In terms of complexity - length, formal structure, and vocabulary - the teachers deemed them similar to the materials usually provided in class. For each text, a fill-in-the-gap test was prepared to be used in the study session. Each of these tests was made of 12 to 14 fill-in-the gap sentences and each sentence had 1 to 4 gaps which could be filled with 1 to 3 words. For each text, an open-questions test with 12 to 16 questions was prepared for the final learning assessment. Correct answers to each of these questions could

require either one word or a short sentence and two blank rows were provided for responding. To mitigate potential ceiling effects, all tests were made slightly more challenging than those typically administered to 5th-grade students. All materials were printed in font Calibri 16, line-spacing 1.15, as suggested by the teachers of the two classes.

**1.3. Procedure.** In all phases (1, 2, 3 and 4), students received a separate sheet for each step of the procedure: text, fill-in-the-gap test for the study session (in the experimental condition only), corrective feedback for self-evaluation (in the experimental condition only) and final evaluation test. Each phase was structured as follows:

- STEP 1: the students received the text and were instructed to carefully read it twice; the text was then withdrawn.
- STEP 2: after 1 (Short Interval condition) or 4 days (Long Interval condition) from STEP 1, participants could study the material according to the condition they were assigned:
    - Participants in the Re-reading condition were given the text back and could re-read it as many times as they wanted within a time limit of 30 minutes. The text was then withdrawn.
    - Participants in the Testing condition were given a fill-in-the-gap test to fulfil in a total of 30 minutes maximum. After the first attempt, the students received a sheet with the correct answers. They were instructed to self-evaluate their answers, marking any errors they found. The teachers then withdrew the correction sheets and students could try to answer the question again on the same sheet they used before, using a pen of a different color. The correction sheet was then given back to the students, and so on. The entire procedure was repeated up to three times within the time limit: in all cases, this allowed students to reach complete accuracy by the end of the session. Teachers were instructed not to provide any suggestions to the students, except for assisting

them in better understanding the meaning of the questions or any unfamiliar words found in the texts.

- STEP 3: Seven days after STEP 2, the final test was administered. During this phase, students were given a time limit of 10 minutes to answer the open-questions test, without access to the original text. The session ended when the teachers collected the answer sheets. After the final test, the teachers were free to provide oral feedback to the students, sharing the correct answers. This supplementary step (i.e., Step 4) was not considered part of the experiment.

**1.4. Scoring.** Before analyzing the data, we rapidly checked the fill-in-the-gap tests, to ensure the accuracy and integrity of the self-evaluation procedure. All students reached complete accuracy in completing the gaps, thus demonstrating that the complexity of the materials was appropriate for their grade. Then, the final open-question tests were corrected by an evaluator (L.F.) who was blind regarding the procedure used by the respondents to study the materials. This blind correction process helped to maintain objectivity and minimize potential bias in the evaluation of the students' answers. For incorrect or missing answers, a score of 0 was assigned. Correct answers were assigned either 0.5 or 1 point, depending on the complexity of the question (i.e., easy or difficult). Partially correct answers were assigned 0 and 0.5 points in the case of easy and difficult questions, respectively. The highest score a student could obtain was 12 points for Tests 1, 2 and 3 and 13 points for Test 4; final scores were converted to percentage value.

**1.5. Additional analyses.** For Phases 1 and 2 we also conducted a mixed ANOVA with Learning Condition as a within-participant factor and Order of the conditions (Re-reading + Short Interval first vs. Testing + Long Interval first) as a between-participant factor. The main effect of Learning Condition proved significant, $F(1, 25) = 41.42$, $p < .001$, $\eta 2 = .154$, but neither the main effect of Order nor the interaction of the two factors did, $Fs < 1$. Since the order in which the two learning conditions were assigned was confounded with the class (5A vs. 5B) to which participants belonged, the analysis also indicates no significant differences between the two classes. This suggests that the

observed effect of the learning condition was genuine and not to be attributed to the specific class of the students. The absence of a significant interaction also demonstrates that the advantage of the Testing + Long Interval condition over the Re-reading + Short Interval condition was the same regardless of the order of these conditions, that is, of whether they were assigned to participants in Phase 1 or 2. This also implies that the specific materials (texts, fill-in-the-gap test and final tests) used in the two phases were equivalent in terms of complexity, although the content was different.

## 2. ORIGINAL MATERIALS

This section contains the original materials (in Italian) of all the four phases (texts, fill-in-the-gap tests for the study sessions, open-question tests for the final tests). As reported in the Scoring section, final evaluation tests of Phases 3 and 4 contained both 0.5 and 1 points questions. The questions worth maximum 0.5 points are indicated with *.

### 2.1. Materials of Phase 1

**Text**

*La civiltà greco-ellenistica (II secolo a. C.)*

A nord della Grecia si estendeva il Regno di Macedonia, con capitale Pella. Sebbene popolato da genti di stirpe ellenica (greca), era rimasto estraneo alle vicende della Grecia.

Gli abitanti praticavano l'agricoltura, la caccia e l'allevamento dei cavalli. Non avevano sviluppato la navigazione perché le loro coste, affacciate sul mare Egeo, erano paludose.

I Macedoni erano un popolo molto unito e disciplinato, governato da un re. Nel 359 a. C. salì al trono il giovane Filippo II. Era stato educato in Grecia e durante quel periodo aveva potuto constatare la debolezza del popolo greco. Era una terra devastata da guerre fratricide (tra persone della stessa nazione) e Filippo II decise di conquistarla.

Per prima cosa Filippo II riorganizzò l'esercito, introducendo lo schieramento a falange, una formazione di fanti disposti su otto file che si proteggevano a vicenda con gli scudi ed erano armati di lance lunghe fino a 7 metri. In questo modo la falange risultava imbattibile. Filippo riorganizzò anche la cavalleria, che aveva il compito di inseguire il nemico sconfitto dalla falange.

Con questo forte esercito il re macedone affrontò i Greci (che non utilizzavano né la falange né la cavalleria) e li sconfisse in una famosa battaglia combattuta a Cheronea nel 338 a. C. Filippo non si comportò con crudeltà nei confronti dei vinti poiché era un sincero ammiratore della civiltà greca.

Proclamò la Lega Panellenica, ossia l'unione di tutti i Greci sotto il suo comando. Sognava inoltre di estendere il suo dominio sui Persiani e si preparava a condurre una spedizione contro di loro, quando venne misteriosamente ucciso.

Il suo progetto venne ripreso dal figlio Alessandro. Sebbene avesse solo vent'anni quando succedette al padre come re macedone, era già esperto di guerra e di governo perché aveva sempre affiancato Filippo II.

Alessandro era stato istruito dai migliori maestri greci, amava la scienza medica e la letteratura, aveva un fisico eccellente e uno straordinario coraggio. Era un ottimo cacciatore e un abile arciere e sopportava ogni fatica insieme ai suoi soldati, dai quali era amatissimo.

L'esercito di Alessandro era composto da uomini esperti, ma era meno numeroso di quello dei Persiani. L'esercito persiano era formato in gran parte da mercenari, soldati che combattevano per denaro e che quindi non erano sempre fedeli al loro capo (il termine deriva da "merces", che significa "denaro").

Alessandro riuscì a vincere i persiani più volte e infine li sconfisse definitivamente nella battaglia di Isso. Per sottrarsi alla cattura, Dario III, re dei persiani, fuggì lasciando la sua famiglia nelle mani dei vincitori. Alessandro, con generosità, la liberò.

Alessandro non si limitò alla conquista della Persia; con una serie di spedizioni occupò il territorio della Fenicia e l'intero Egitto, dove fondò una città che prese il suo nome, Alessandria. Tale città divenne famosa per due grandi costruzioni. Una era la biblioteca in cui erano custodite 700000 opere scritte. L'altra era il primo faro del mondo, una torre di marmo alta 120 metri che aveva sulla sommità un grande fuoco sempre acceso per far luce ai marinai. Esso rimase in piedi fino al 1.300 d. C. quando fu distrutto da un terremoto.

Alessandro si spinse poi in Oriente fino alle rive del fiume Indo. Il suo impero si estendeva così su tre continenti.

Alessandro fu il più grande conquistatore dell'antichità e per questo viene chiamato "Magno", cioè "Grande". Nel 323 a.C., a soli ventitré anni, morì per una febbre malarica. Anche se morì giovane, il suo sogno si realizzò: egli riuscì a unire popoli diversi ponendo fine alle loro guerre interne e a portare gli influssi della civiltà greca. Dall'unione di queste civiltà nacque quella che gli storici hanno chiamato civiltà Ellenistica (dal termine "Elleni" = Greci).

Dopo la sua morte il vasto impero si smembrò a causa delle lotte scoppiate tra i suoi generali che aspiravano alla successione. Essi crearono Stati diversi, detti Regni Ellenistici. I più importanti furono quelli di Macedonia, di Siria e d'Egitto.

## Fill-in-the-gap test (for the study session)

*Completa le seguenti frasi inserendo le parole mancanti.*

1. Il regno di Macedonia si trovava più a ........... rispetto alla ............. e aveva come capitale la città di ...............

2. Le attività più importanti dei macedoni erano tre: ..................., ................... e ................... Non praticavano invece la ....................
3. Il primo importante re macedone fu ................. che, appena salito al trono, decise di conquistare il popolo dei ..............
4. Il re rinforzò l'esercito e introdusse due importanti innovazioni: lo schieramento a .................. e la ..................
5. Nella battaglia di Cheronea tra ................. e ........... i vincitori furono i .................., che trattarono con gentilezza gli sconfitti.
6. Il re macedone fondò la .......... ....................., che riuniva tutti gli abitanti della .............. sotto il suo potere.
7. Quando il re macedone fu ucciso, salì al trono il suo giovane figlio ..................., che portò avanti i progetti del padre, dal quale aveva imparato a governare e a ........................
8. L'esercito del nuovo re macedone era .......... numeroso di quello dei ................
   Il nuovo re macedone era molto amato dai suoi soldati mentre l'esercito dei ............... era formato soprattutto da ..................., soldati che combattevano solo per .......... e che non erano così fedeli.
9. I macedoni sconfissero definitivamente i ............... nella battaglia di Isso. Successivamente conquistarono altri due importanti territori, la ............. e l'intero ..............
10. Il nuovo re macedone fondò la città di ........................, che aveva due edifici importanti: la ....................... e il ..............
11. Il nuovo re macedone, infine, spostò il suo interesse anche sull'...................
    Il suo impero giunse così ad occupare ....... continenti.
12. Il nuovo re macedone venne chiamato .............., cioè .................
    Nei suoi pochi anni di regno riuscì a realizzare il suo sogno di unire popoli diversi accomunati dalla cultura .............., fondando così la civiltà .................
    13. Dopo la sua morte il suo impero si divise e i suoi successori crearono stati diversi, detti ......... ..................

**Corrective feedback (for self-evaluation)**

1. nord, Grecia, Pella
2. agricoltura, caccia, allevamento, navigazione
3. Filippo II, greci
4. falange, cavalleria
5. macedoni, greci, macedoni
6. Lega Panellenica, Grecia
7. Alessandro, combattere (va bene anche "fare la guerra")
8. meno, persiani, persiani, mercenari, soldi
9. persiani, Fenicia, Egitto
10. Alessandria, biblioteca, faro
11. Oriente, tre
12. Magno, Grande, greca, Ellenistica
13. Regni Ellenistici

**Final evaluation test**

*Rispondi in modo breve alle seguenti domande.*

1. Dove si trovava il regno di Macedonia?
2. Quali erano i mestieri più diffusi tra i macedoni?

3. Come si chiamava il primo re macedone?
4. Quali furono i due importanti cambiamenti che portò egli nell'esercito?
5. Da chi era composta la Lega Panellenica?
6. Chi fu il giovane successore del primo re macedone?
7. Come si chiamavano i soldati che facevano parte dell'esercito persiano e cosa significa la parola "merces"?
8. In Egitto, il giovane re macedone fondò un'importante città. Come la chiamò?
9. Cosa fece costruire egli nella nuova città?
10. Come venne chiamato il giovane re e cosa significa questa parola?
11. Qual era il sogno che il giovane re riuscì a realizzare nei suoi pochi anni di regno?
12. Cosa accadde all'Impero dopo la sua morte e come vennero chiamati i nuovi stati fondati?

## 2.2. Materials of Phase 2

### Text

*La civiltà degli etruschi (VII-V secolo a. C.)*

La civiltà degli Etruschi si sviluppò in Italia verso la metà del IX secolo a.C. ma il periodo di massimo splendore fu tra il VII e il V secolo a.C., quando, secondo gli storici, gli Etruschi erano circa 400 000.

La terra in cui abitavano, detta Etruria, andava dall'Arno al Tevere e dagli Appennini al mar Tirreno. Oltre a questa grande area, gli Etruschi avevano occupato un territorio che comprendeva gran parte della Pianura Padana (Etruria padana). Possedevano inoltre un'area che si estendeva dal golfo di Napoli fino a Salerno e confinava con le colonie greche (Etruria campana).

Gli Etruschi erano un popolo di abili contadini e mercanti. Bonificarono le paludi per poterle coltivare e costruirono importanti opere idrauliche: canali per irrigare i campi e dighe per ottenere bacini di acqua dolce. Ottenevano raccolti molto abbondanti e vendevano i loro prodotti anche ai popoli vicini.

Le città etrusche sorgevano vicino alle rive dei fiumi o del mare, dove vennero fondati importanti porti e scali commerciali. Nelle città marittime era molto praticata la pesca, soprattutto di tonni.

Gli etruschi furono il primo popolo italico a costruire vere e proprie città con mattoni e mura di cinta. Venivano fondate sulla sommità di colline, per essere più facilmente difendibili, e nel punto più in alto sorgeva il tempio. Le piante delle città erano regolari e le vie principali erano il cardo (che attraversava la città da nord a sud) e il decumano (da est a ovest).

La più importante invenzione etrusca fu l'arco con chiave di volta, che venne poi usato anche dai Romani.

La civiltà etrusca venne condizionata molto dalla civiltà greca. Gli Etruschi entrarono in contatto con i Greci grazie ai commerci e vennero influenzati dalla loro cultura, tanto da imitare la loro organizzazione sociale. Gli Etruschi, infatti, si organizzarono in città-stato indipendenti e autonome le une dalle altre. Le principali furono Tarquinia, Populonia, Orvieto, Cerveteri, Veio, Vulci, Perugia, Arezzo e Volterra. Gli abitanti delle diverse città avevano tradizioni e stili di vita simili e fecero alleanze e patti commerciali.

Ogni città aveva un re chiamato *lucumone* che era a capo dell'esercito e dei sacerdoti e veniva scelto tra le famiglie nobili. I nobili possedevano la maggior parte della terra, erano guerrieri e occupavano le cariche più importanti nell'esercito. Gli schiavi, gli operai, i contadini e gli artigiani costituivano la maggioranza del popolo.

Con il passare degli anni le famiglie dei ricchi proprietari terrieri e dei commercianti guadagnarono sempre più potere e fondarono un governo di tipo oligarchico come quello di Sparta.

Gli Etruschi parlavano in lingua etrusca e per scrivere usavano i segni dell'alfabeto greco, che incidevano su lamine di metallo e su vasi. I pochi che sapevano scrivere appartenevano alle classi sociali più elevate. Ancora oggi gli storici non sono riusciti a decifrare completamente la scrittura etrusca, che in parte rimane misteriosa.

Gli Etruschi erano politeisti. Anche nella religione furono influenzati dei Greci e molte delle loro divinità avevano le stesse caratteristiche degli Dei greci, seppur con nomi diversi.

Secondo gli Etruschi ogni uomo aveva un destino già scritto e non modificabile, che veniva deciso dagli Dei. Per comunicare con gli uomini gli Dei usavano dei segnali naturali, che solo i sacerdoti sapevano interpretare.

Gli etruschi credevano che la morte fosse solo il passaggio verso una nuova vita, per questo curavano molto la sepoltura. Costruivano delle vere e proprie case per defunti per la loro nuova vita nell'aldilà. Un po' alla volta nacquero delle vere e proprie città chiamate "necropoli", che letteralmente significa "città dei morti".

Lo studio delle necropoli è stato molto importante perché ci ha permesso di capire la vita quotidiana degli Etruschi. Grazie ai reperti trovati dagli archeologi, infatti, si è scoperto che erano abilissimi artigiani e scultori. Lavoravano il bronzo, l'oro e la terracotta e realizzavano affreschi e dipinti. Molti degli oggetti raffinati che producevano venivano usati per il commercio.

## Fill-in-the-gap test (for the study session)

*Completa le seguenti frasi inserendo le parole mancanti.*

1. La civiltà etrusca nacque in ................ a metà del IX secolo a.C. La terra degli etruschi era chiamata .................
2. Possedevano anche altri due territori, la Pianura Padana, che chiamavano ............... ................. e un'area del sud Italia detta ................ ...................
3. Gli etruschi erano un popolo di esperti contadini e ......................
   Costruirono grandi opere .........................., tra cui canali per ....................................... e ................... per conservare l'acqua dolce.
4. Le città sorgevano vicino ai fiumi o al mare, dove gli Etruschi fondarono importanti .................... per ............................
5. Nel punto più in alto di ogni città sorgeva il ..................... e le due vie principali erano il ................ (che andava da nord a sud) e il ..................... (da est a ovest).
6. Gli etruschi inventarono l'............... con chiave di volta, che venne in seguito usato anche dai ...............
7. La civiltà che più influenzò gli etruschi fu quella .....................
   Seguendo il loro esempio, gli etruschi divisero il regno in tante .............-............ che erano ........................ e autonome le une dalle altre.

8. Il governo di ogni città era affidato a un re detto ..................., che era a capo dell’.................. e dei ..................... e veniva scelto tra i ...................
9. Con il tempo le famiglie dei proprietari terrieri e dei ...................... diventarono sempre più potenti, fondando un governo di tipo ..................... come a Sparta.
10. La lingua parlata dagli etruschi era l’................, ma per scrivere usavano l’alfabeto ..................
11. Gli etruschi erano ..................... (=adoravano molti dei) e, anche nella religione, furono influenzati molto dalla civiltà .................
12. Gli etruschi credevano che il .................. di ogni uomo fosse già scritto e deciso dagli .................., che comunicavano con gli uomini attraverso dei segnali naturali che solo i ................... sapevano interpretare.
13. Per gli etruschi la morte era solo un ..................... verso una nuova vita, per questo si preoccupavano molto della ................... dei loro cari. Costruivano delle ............... per i defunti e con il tempo nacquero delle vere e proprie “città dei morti”, che venivano chiamate ...................... . I ................ trovati al loro interno hanno permesso di capire molto sulla vita quotidiana degli etruschi.

## Corrective feedback (for self-evaluation)

1. Italia, Etruria
2. Etruria Padana, Etruria Campana
3. mercanti (va bene anche commercianti), idrauliche (va bene anche architettoniche), irrigare i campi (va bene anche dare acqua ai campi), dighe (va bene anche bacini)
4. porti (va bene anche scali), il commercio (va bene anche commerciare)
5. tempio, cardo, decumano
6. arco, Romani
7. greca (va bene anche dei Greci), città-stato, indipendenti
8. lucumone, esercito, sacerdoti, nobili
9. mercanti (va bene anche commercianti), oligarchico
10. etrusco, greco (va bene anche dei Greci)
11. politeisti, greca (va bene anche dei Greci)
12. destino (va bene anche futuro), dei, sacerdoti
13. passaggio, sepoltura, case, necropoli, reperti (va bene anche manufatti)

## Final evaluation test

*Rispondi in modo breve alle seguenti domande.*

1. Come si chiamava la porzione di penisola italiana abitata dagli Etruschi?
2. Quali erano i mestieri più diffusi tra gli Etruschi?
3. Per coltivare la terra gli Etruschi costruirono imponenti opere idrauliche. Quali?
4. Vicino a cosa venivano costruite le città etrusche? Perchè?
5. Cos’erano il “cardo” e il “decumano”?
6. Gli Etruschi furono molto influenzati da un’altra importante civiltà. Quale?
7. Che caratteristiche avevano le città-stato etrusche?
8. Chi era il lucumone? Tra chi veniva scelto?
9. Con il tempo il potere passò nelle mani dei ricchi mercanti e proprietari terrieri. Si creò quindi un governo di che tipo?

10. Da chi era deciso il destino degli uomini, secondo gli Etruschi?
11. Che cos’era la morte per gli Etruschi?
12. Cos’erano le “necropoli”? Perché è stato così importante studiarle?

## 2.3. Materials of Phase 3

### Text

*Le origini di Roma - La realtà storica*

Nel IX secolo a.C., nei territori dell’Italia centrale che oggi chiamiamo Lazio, vivevano varie tribù di pastori e agricoltori: Latini, Sabini e gruppi di Etruschi.

Quell’area era prevalentemente collinare e i colli principali erano sette: il Palatino, l’Aventino, il Celio, l’Esquilino, il Quirinale, il Viminale, il Campidoglio.

I popoli della zona avevano costruito i loro villaggi sulle colline perché la pianura era paludosa e perché il rischio di inondazioni era alto. Inoltre, in questo modo, i loro villaggi erano più facilmente difendibili. Non avevano infatti costruito cinte murarie per proteggersi dai nemici.

In pianura scorreva il fiume Tevere, che era un’importante via di comunicazione e permetteva lo scambio delle merci. Il prodotto più importante era il sale, che veniva estratto nelle saline alla foce del Tevere ed era fondamentale per la conservazione dei cibi.

In mezzo al fiume, vicino al colle Palatino, si trovava l’isola Tiberina, che permetteva l’attraversamento del fiume (a nuoto o su zattere). L’isola era un punto strategico per l’incontro tra i popoli che vivevano a nord e quelli che vivevano a sud.

Nell’VIII secolo a.C. i villaggi, che si erano ingranditi e rafforzati, decisero di unirsi e di fondare un’unica città. La nuova città prese il nome di Roma, dal nome della tribù Ramnes che abitava sul colle Capitolino.

*La leggenda*

La leggenda narra che la città di Roma fu fondata il 21 aprile 753 a.C. da Romolo, sul colle Palatino.

Romolo e il suo gemello Remo erano figli del dio Marte e della sacerdotessa Rea Silvia, figlia del re di Albalonga, un'importante città della zona. Il fratello del re, per ereditare il trono al posto di Romolo e Remo, ordinò di ucciderli.

I servi ebbero pietà dei due piccoli, li misero in una cesta e li abbandonarono alla corrente del Tevere. Il fiume trascinò la cesta fino a una grotta che si trovava alla base del colle Palatino. Qui i gemelli furono trovati e allattati da una lupa. Poi furono allevati e cresciuti da due pastori.

Divenuti grandi, Romolo e Remo scoprirono le loro nobili origini e decisero di costruire una nuova città. Subito cominciarono a litigare per dove fondarla e così si affidarono al fato (al destino) secondo la tradizione etrusca. Alla fine, ebbe la meglio Romolo, che scelse il colle Palatino.

Remo scavalcò il tracciato della nuova città, comportandosi come un nemico invasore, e per questo fu ucciso dal fratello. Romolo divenne così il primo re della città, che da lui prese il nome Roma.

*Il periodo della monarchia*

Il governo di Roma fu inizialmente di tipo monarchico. Negli anni si succedettero al trono diversi re, ma secondo la tradizione i più importanti sono stati sette:

- ROMOLO: fu il fondatore e il primo re e diede alla città la prima Costituzione
- NUMA POMPILIO: fece istituire il calendario diviso in dodici mesi per stabilire i giorni lavorativi e quelli delle più importanti festività
- TULLO OSTILIO: fu un re guerriero e conquistò la città rivale di Albalonga
- ANCO MARZIO: ingrandì la città e fece terminare la costruzione del primo ponte in legno sul Tevere
- TARQUINIO PRISCO: introdusse alcune tradizioni etrusche e fece costruire il primo sistema di fognature per scaricare nel Tevere le acque sporche
- SERVIO TULLIO: fece costruire una nuova cinta muraria e divise i cittadini in cinque classi sociali a seconda del censo (della ricchezza)
- TARQUINIO IL SUPERBO: fu considerato un tiranno e fu cacciato da una rivolta popolare nel 509 a.C. Con lui terminò il periodo della monarchia.

## Fill-in-the-gap test (for the study session)

*Completa le seguenti frasi inserendo le parole mancanti.*

1. Nel XI secolo a.C. il ................ era abitato da tre tribù: ................, .................... e .................. Quella regione era per lo più collinare e c'erano in tutto ................. colli.
2. I villaggi **non** erano circondati da ................ e venivano costruiti sulle .................... per proteggersi dagli attacchi nemici e dalle frequenti .........................
3. Il fiume ................... era un'importante via di ....................... e di .................... di merci. Il prodotto principale era il .................. L'isola .................... permetteva di attraversare il fiume da una parte all'altra.
4. Nel tempo i villaggi diventarono sempre più grandi e decisero di .................... e di fondare un'unica ................, che chiamarono .................
5. Secondo la leggenda la città di Roma fu fondata nel 753 a.C. da .................... sul colle ....................
6. I gemelli .................. e .................. erano figli del dio ................. e della sacerdotessa Rea Silvia. Da piccoli furono abbandonati in una cesta che venne gettata nel fiume .................... I due bambini furono trovati e allattati da una ................. e poi furono cresciuti da due ..................... Una volta adulti decisero di .................. una nuova città, che deve il suo nome al gemello vincitore: .................
7. All'inizio la città di Roma ebbe un governo di tipo ..................... e i re principali furono sette: .................., Numa Pompilio, Tullio Ostilio, Anco Marzio, Tarquinio Prisco, Servio Tullio e ..................................

8. ................. fu il primo re e diede alla città la sua prima .......................
9. Numa Pompilio introdusse il .................... per stabilire i giorni di lavoro e quelli di festa.
10. Tullio Ostilio era un re ................... e conquistò la città di Albalonga.
11. Anco Marzio fece costruire il primo ................. sul fiume ...................
12. Tarquinio Prisco fece costruire le prime .................... per le acque sporche.
13. Servio Tullio divise i cittadini in cinque classi sociali sulla base del .................
14. .................................. si comportò da .................. e fu cacciato con una rivolta popolare nel 509 a.C. Con lui finì il periodo della monarchia.

**Corrective feedback (for self-evaluation)**

1. Lazio, Latini, Sabini, Etruschi, sette
2. mura (o cinte murarie), colline, inondazioni (o esondazioni)
3. Tevere, comunicazione, scambio (o commercio), sale, Tiberina
4. unirsi (o allearsi), città, Roma
5. Romolo, Palatino
6. Romolo, Remo, Marte, Tevere, lupa, pastori, fondare (o costruire), Romolo
7. monarchico, Romolo, Tarquinio il Superbo
8. Romolo, Costituzione
9. calendario
10. guerriero
11. ponte, Tevere
12. fognature
13. censo (o ricchezza)
14. Tarquinio il Superbo, tiranno (o dittatore)

**Final evaluation test**

*Leggi attentamente e rispondi in modo breve alle seguenti domande.*

1. Quali erano le tre tribù che abitavano il territorio che oggi chiamiamo Lazio?
2. Quanti erano i colli della zona? Perché i villaggi venivano costruiti sulle colline?
3. Come si chiamava il fiume che scorreva in quelle terre? Perchè era importante? Qual era il prodotto principale per il commercio?
4. Da chi e dove fu fondata la città di Roma secondo la leggenda?
5. Come si chiamavano i due gemelli della leggenda? Da chi furono trovati e allattati all'inizio? E poi chi li allevò?
6. Che tipo di governo ebbe all'inizio la città di Roma? Quanti furono i re principali?
7. Come si chiamava il primo re e che cosa diede di importante alla città?
8. Quale novità introdusse il re Numa Pompilio per stabilire i giorni di lavoro e quelli di festa?
9. Cosa fece il re guerriero Tullio Ostilio alla città nemica di Albalonga? *
11. Che cosa fece costruire sul fiume il re Anco Marzio? *
12. Che cosa fece costruire il re Tarquinio Prisco per raccogliere le acque sporche?
13. Sulla base di quale caratteristica dei cittadini il re Servio Tullio li divise in classi sociali? Quante erano le classi?
14. Chi fu l'ultimo re di Roma? Perché fu cacciato dalla città?

## 2.4. Materials of Phase 4

**Text**

*La società romana*

La società romana era divisa in patrizi e plebei (oltre agli schiavi).

I patrizi erano i discendenti delle famiglie che formarono il primo nucleo di Roma. Erano ricchi proprietari terrieri e godevano dei maggiori privilegi. Partecipavano al governo e all'amministrazione dello Stato, sedevano in Senato e nominavano i consoli. Essi potevano sposare solo donne patrizie.

I plebei, che rappresentavano la maggioranza della popolazione, erano cittadini liberi, ma non partecipavano al governo della città. Erano contadini, artigiani e mercanti e a loro spettava lo svolgimento della maggior parte dei lavori. Infatti, costruivano gli edifici, gestivano i negozi e difendevano la città dagli attacchi nemici prestando servizio nell'esercito.

I patrizi e i plebei si distinguevano anche dall'abbigliamento. I patrizi indossavano un mantello sopra alla tunica e portavano anelli d'oro alle dita. Le donne patrizie sfoggiavano anche orecchini, collane e bracciali. I plebei, invece, indossavano delle semplici tuniche strette in vita da una cintura.

La "domus" era la tipica casa dei patrizi, quasi sempre a un piano e a pianta rettangolare o quadrata. Nella parte centrale c'era un'apertura sul tetto da dove entravano la luce e l'acqua piovana, che si raccoglieva in una vasca.

I plebei, invece, abitavano nelle "insulae", affollatissimi edifici dai tre ai cinque piani. Gli appartamenti erano piccolissimi, spesso formati da una sola stanza, scomodi, bui e senza acqua.

Le "villae" erano grandi residenze di campagna di proprietà dei romani più ricchi. Avevano tante stanze, pavimenti in marmo, colonne, giardini, piscine e anche un grande parco.

*Il periodo della Repubblica*

Dopo la cacciata del re Tarquinio il Superbo (509 a.C.), Roma diventò una Repubblica (dal latino "res publica" = cosa pubblica): non fu più governata da un re, ma dal SENATO e da DUE CONSOLI.

I consoli restavano in carica un anno e potevano essere eletti al massimo due volte di seguito. Venivano scelti solo tra i patrizi, per questo Roma era definita una Repubblica "aristocratica". I consoli avevano il comando dell'esercito, mettevano in pratica le leggi e convocavano il Senato.

Il Senato era costituito da 300 patrizi, controllava l'operato dei consoli e veniva consultato prima di far passare le leggi.

Al di sotto dei consoli si trovavano altri magistrati:

- i censori, che calcolavano le tasse da far pagare ai cittadini;
- i questori, che gestivano il denaro dello Stato;
- i pretori, che amministravano la giustizia;
- gli edili, che si occupavano della costruzione di strade ed edifici.

In caso di guerra veniva nominato un dittatore, che aveva pieni poteri e rimaneva in carica solo sei mesi.

Dato che solo i patrizi avevano un ruolo nel governo della città, i plebei organizzarono una protesta per far valere i propri diritti. Fu una specie di sciopero, durante il quale abbandonarono la città, ritirandosi sul colle Aventino. I plebei chiusero tutte le botteghe, si rifiutarono di combattere e lasciarono Roma senza protezione militare.

Con la loro protesta i plebei ottennero delle leggi scritte e il diritto di eleggere due rappresentanti al Senato, i “tribuni della plebe”, che dovevano difendere i loro interessi.

Le prime leggi scritte furono incise su 12 tavole di bronzo ed esposte nella piazza principale della città, perché tutti potessero vederle. Si basavano sul principio dell’uguaglianza di tutti i cittadini di fronte alla legge.

Con il tempo i plebei e i patrizi più benestanti costituirono una nuova classe politica, la “nobilitas”. Nacque così il Popolo romano come unità politica, come indica la famosa espressione “Senatus Populusque Romanus” (SPQR) = il Senato e il Popolo romano.

La Repubblica rimase la forma di governo di Roma per quasi cinque secoli.

**Fill-in-the-gap test (for the study session)**

*Completa le seguenti frasi inserendo le parole mancanti.*

1. La società romana era divisa in 3 classi sociali: ........................., ......................... e .........................
2. I ......................... erano ricchi proprietari terrieri, partecipavano al ......................... dello Stato, sedevano in Senato e nominavano i .........................
3. I ......................... erano la ......................... della popolazione, erano cittadini ......................... ma non avevano alcun ruolo nel governo. Erano per lo più contadini, ......................... e artigiani e difendevano la città dagli attacchi nemici.
4. I patrizi vivevano nelle ......................... mentre i plebei abitavano nelle ......................... Le ......................... erano le grandi residenze di campagna dei romani più ricchi.
5. Dopo la cacciata dell’ultimo re ......................... il Superbo, Roma divenne una Repubblica (dal latino “res publica” = ..................................................), governata dal ......................... e da due ........................., che venivano scelti solo tra i ......................... Per questo motivo Roma fu definita una Repubblica .........................
6. I consoli avevano il compito di comandare l’........................., mettere in pratica le ......................... e convocare il .........................
7. Il Senato era costituito da 300 ........................., controllava l’operato dei ......................... e veniva consultato prima di approvare le .........................
8. Gli altri magistrati erano: ........................., che calcolavano le tasse, ........................., che gestivano il denaro dello Stato, ........................., che amministravano la giustizia, ........................., che dirigevano la costruzione di strade ed edifici. Un’altra figura importante era il ........................., che veniva nominato in caso di .........................

9. Per far valere i propri diritti, i .......................... organizzarono una .......................... Ottennero così delle .......................... scritte e il diritto di eleggere due rappresentanti, i .................................................., che dovevano difendere i loro interessi in Senato.
10. Le prime leggi scritte vennero esposte nella .......................... principale della città perché tutti potessero vederle. Si basavano sul principio che tutti i cittadini sono .......................... di fronte alla legge.
11. Con il tempo i romani più ricchi formarono una nuova classe detta .........................., che rappresentava il .......................... romano, come indica l'espressione "Senatus Populusque Romanus" (SPQR) = Il Senato e il .......................... romano.
12. Roma rimase una Repubblica per quasi .......................... secoli.

**Corrective feedback (for self-evaluation)**

1. patrizi, plebei, schiavi
2. patrizi, governo (o amministrazione), consoli
3. plebei, maggioranza (o maggior parte), liberi, mercanti (o commercianti)
4. domus, insulae, villae
5. Tarquinio, cosa pubblica, Senato, consoli, patrizi, aristocratica
6. esercito, leggi, Senato
7. patrizi, consoli, leggi
8. censori, questori, pretori, edili, dittatore, guerra
9. plebei, protesta (o rivolta o sciopero), leggi, tribuni della plebe
10. piazza, uguali
11. nobilitas, Popolo, Popolo
12. cinque

**Final evaluation test**

*Leggi attentamente e rispondi in modo breve alle seguenti domande.*

1. Quali erano le 3 classi in cui era divisa la società romana?
2. Qual era l'unica classe che partecipava al governo della città? *
3. Quale classe rappresentava la maggioranza della popolazione? *
4. Come si chiamavano le case dei patrizi? E quelle dei plebei?
5. Quale forma di governo assunse Roma dopo la cacciata dell'ultimo re Tarquinio il Superbo? Cosa significa "res publica"?
6. Da chi era governata la Repubblica di Roma?
7. Perché la Repubblica di Roma venne definita "aristocratica"?
8. Qual era uno dei compiti dei consoli?
9. Che cosa facevano i censori? *
10. Che cosa facevano gli edili? *
11. Quando veniva nominato il dittatore? *
12. Che cosa fecero i plebei per far valere i loro diritti?
13. Chi erano i tribuni della plebe?
14. Su quale principio si basavano le prime leggi scritte?
15. Cosa significa Senatus Popolusque Romanus (SPQR)?
16. Per quanti secoli durò la Repubblica di Roma? *
